# The self and conscious experience

**DOI:** 10.3389/fpsyg.2024.1340943

**Published:** 2024-01-25

**Authors:** Giorgio Marchetti

**Affiliations:** Mind, Consciousness and Language Research Center, Alano di Piave, Italy

**Keywords:** conscious experience (CE), self (S), self/non-self distinction, attention, organ of attention

## Abstract

The primary determinant of the self (S) is the conscious experience (CE) we have of it. Therefore, it does not come as a surprise that empirical research on S mainly resorts to the CE (or lack of CE) that subjects have of their S. What comes as a surprise is that empirical research on S does not tackle the problem of how CE contributes to building S. Empirical research investigates how S either biases the cognitive processing of stimuli or is altered through a wide range of means (meditation, hypnosis, etc.). In either case, even for different reasons, considerations of how CE contributes to building S are left unspecified in empirical research. This article analyzes these reasons and proposes a theoretical model of how CE contributes to building S. According to the proposed model, the phenomenal aspect of consciousness is produced by the modulation—engendered by attentional activity—of the energy level of the neural substrate (that is, the organ of attention) that underpins attentional activity. The phenomenal aspect of consciousness supplies the agent with a sense of S and informs the agent on how its S is affected by the agent’s own operations. The phenomenal aspect of consciousness performs its functions through its five main dimensions: qualitative, quantitative, hedonic, temporal, and spatial. Each dimension of the phenomenal aspect of consciousness can be explained by a specific aspect of the modulation of the energy level of the organ of attention. Among other advantages, the model explains the various forms of S as outcomes resulting from the operations of a single mechanism and provides a unifying framework for empirical research on the neural underpinnings of S.

## Introduction

The primary determinant of our intuition of the self (from now on S) as a unitary, sentient entity differentiated from other entities, which is temporally extended and is provided with its own perspective from which it interacts with what surrounds it, is our *conscious experience* (or *feeling* or *sense*; from now on CE) of S.

The close dependence of S on CE has been observed by various philosophers and scholars, to mention just a few: Locke (1689/1917), Sartre (1956), Damasio (1998), Zahavi (2005), Humphrey (2006), Legrand and Ruby (2009), Gallagher (2012), Marchetti (2012a, 2022), Demertzi et al. (2013), Berkovich-Ohana and Glicksohn (2014), Klein (2014), Velmans (2014), Lou et al. (2017), Nida-Rümelin (2017), and Kiverstein (2020). For example, Locke (1689/1917, Book II, Ch. 27, Sec. 23, p. 259) states that “Consciousness alone makes self. Nothing but consciousness can unite remote existences into the same person” and Damasio (1998, p. 1880) states that “Consciousness occurs when we can generate, automatically, the sense that a given stimulus is being perceived in a personal perspective; the sense that the stimulus is *owned* by the organism involved in the perceiving; and, last but not least, the sense that the organism can act on the stimulus (or fail to do so), that is, the sense of *agency*.”

Without CE, it would be impossible for S not only to be described but also to take on its form and most of its features (such as first-person perspective, self/non-self distinction, synchronic and diachronic unity, and so on. See, for example, Williford et al., 2012; Gallagher, 2013), as we experience them. To exist as we know and experience it, S must be actualized and made present in our consciousness. The very existence of S depends on the daily CEs we have of ourselves as agents, individuals, and persons: how we relate to others, the space we occupy, the boundaries of our body, how limited we are in our movements, what we are able or not able to do, how the feelings of effort and pain constrain our activities, the feeling of continuity that we have (I am the same person I was 10 years earlier, and I will be the same person also in future), the feeling that our experiences belong to us and not to someone else, how our perspective differs from other people’s perspective, and so on. If we did not experience all this, it would be impossible for us to know who we are, what we can and cannot do, and how we differ from others.

The dependence of S on CE becomes even more apparent if one considers that (a) S is not a fixed entity “living” on its own (Fingelkurts et al., 2020) but a property that emerges (already *in utero*: Ciaunica et al., 2021) and develops in time (Rochat, 2003; Cleeremans, 2008), thanks to the experiences the individual continuously undergoes; (b) S is not a thing but a process, which is present at all times when we are conscious (Damasio, 2010, p. 8); and (c) the sense of S is modified and reinforced every time we intentionally reflect on the capacities we have to decide, choose, judge, etc.

The fact that S is primarily determined by CE does not rule out that there can be an unconscious S (or an unconscious part of S: Velmans, 2014; Schaefer and Northoff, 2017). There is ample evidence of an unconscious or preconscious S (Perrin et al., 2006; Geng et al., 2012; Bola et al., 2021). For example, Bola et al. (2021), using a backward masking procedure, show that a self-face image automatically captures attention even when it is processed unconsciously (as opposed to other kinds of faces, e.g., familiar ones, which do not attract attention in the unconscious condition).

However, one should be cautious not to mistake this kind of evidence as indicating the possibility that an unconscious S can develop *without* CE. Actually, what this evidence shows is that S can have an unconscious existence only *once* it has attained a certain form, thanks to CE. Indeed, these experiments use stimuli such as self-faces or self-names that participants must have already consciously processed and learned before the experiments are performed.

Yet, someone could argue (Velmans, 2014, p. 19) that consciousness of a given process does not make consciousness responsible for the operation of that process. While this certainly holds for physical processes (watching paint dry does not actually make it dry), this does not hold for many (albeit not for all) mental processes, least of all for S. Only in and through consciousness can S be actualized, take on a dimension for us, and be continuously modified: should we not experience pain, for example, we will never know our limits, how we react in certain circumstances, that we can be hurt by something, and so on.

The relationship between the material basis of S and CE is quite a different issue. It seems undisputable that S needs a material basis to exist, call it proto-self (Damasio, 1999) or some other way. But can there be a material S without CE, or, in other words, can S exist before any form of CE comes into play? The answer depends very much on how one defines S. Plausible definitions of S that do not involve CE have been provided by several scholars (Legrand, 2004; Llinás and Roy, 2009; Reddy et al., 2019; Woźniak, 2019; Kiverstein, 2020). For example, Legrand (2004) proposes the notion of a purely biological self, such as the immunological system, capable of producing itself without the control of an external agent by remaining open to the external environment. Llinás and Roy (2009) and Reddy et al. (2019) define S as a “centralization of prediction,” that is, as a predicting organ that endows an agent with the capacity to anticipate the outcome of its actions based on incoming and learned stimuli and some inherited abilities. Similarly, Woźniak (2019) defines S as a representational structure in a Bayesian brain. These types of definitions, even though they may lead to disconcerting, counterintuitive conclusions (e.g., if S is a predicting organ, then even robots may possess S) are certainly legitimate. However, they have very limited explanatory power when applied to understanding the human being. They can only account for the part of S that is physically and biologically determined but not for the part of S that continuously emerges due to our daily CEs. That is, they can certainly contribute to explaining how the physical self/non-self boundary forms, but they fall short in explaining how other types (e.g., psychological, social, cultural, economic, political, and ideological) of self/non-self boundaries or other aspects of S (such as the first-person perspective) may form. Only through CE can one explain, for example, how an individual can, unexpectedly, identify with a certain ideology or religion or how an individual can even reject their own physical identity (e.g., xenomelia, see Brugger et al., 2013) or sexual identity. For this reason, in this article, I will exclusively discuss a notion of S that involves CE. More specifically, I will focus primarily on the more basic types of S involving CE, such as the “core self” of Damasio (1999, 2010), assuming—following Damasio (1999, 2010)—that the more complex forms of S, such as the “autobiographical” or “narrative” S, derive from the simpler ones.

Finally, it could be claimed that there are organisms that possess (elementary forms of) CE without S. This is what, for example, Lacalli (2023) maintains, based on an evolutionary account (agency, or S, requires real-time feedback, while this is not required by consciousness). While from a purely evolutionary viewpoint, this is theoretically plausible, I consider it implausible from a functional perspective. As Solms (2019, p. 7) observes, CE is: “the vehicle whereby complex organisms monitor and maintain their functional and structural integrity in unknown situations.” CE primarily achieves this by providing the organism with a condensed (albeit partial) and unified representation of itself, that is, S.

Taking the importance of CE for S, it does not come as a surprise that empirical research on S mainly resorts to the CE (or lack of CE) of participants. What comes as a surprise is that empirical research on S does not tackle the problem of how CE contributes to building S.

Generally speaking, empirical research investigates how S (i) either biases the cognitive processing of stimuli (e.g., Sui et al., 2012a,b, 2013, 2015; Frings and Wentura, 2014; Northoff, 2016; Sui and Humphreys, 2017b; Scalabrini et al., 2022) or (ii) is altered through a wide range of means such as meditation (Berkovich-Ohana et al., 2013; Jerath et al., 2016; Fingelkurts et al., 2016a,b, 2020; Millière et al., 2018), hypnosis (Kallio and Revonsuo, 2003), perceptual deprivation (Glicksohn and Ben-Soussan, 2020), pharmacological means (Millière et al., 2018; Deane, 2020), induced illusory own-body perceptions (Ionta et al., 2011; Petkova et al., 2011; Blanke, 2012; Pfeiffer et al., 2013) and magnetic brain stimulation (Lou et al., 2004; Luber et al., 2012; Dary et al., 2023), or by neurological disorders such as epileptic seizure (Johanson et al., 2008; Blumenfeld, 2011, 2012), or by psychological and psychiatric disorders such as xenomelia (Brugger et al., 2013), or by states of consciousness other than conscious wakefulness (Laureys, 2005) such as sleep dreams (Windt, 2010, 2015) and near-death experience (Vanhaudenhuyse et al., 2009; Martial et al., 2020). In either case, even if for different reasons, empirical research leaves considerations of how CE contributes to building S unspecified.

As will be shown in the section “How the self modulates the neural and cognitive processing of stimuli,” empirical research that investigates how S biases the cognitive processing of stimuli while offering important insights into the possible functions of S and the neural underpinnings of self-related processing, primarily targets S in a particular context (cultural, social, cognitive, synaptic, etc.) rather than S *per se* (Klein, 2014). This implies that, for this type of empirical study, the investigation of how CE contributes to building S is left unexplored, thereby limiting its ability to exhaustively explain the cognitive and neural processes that constitute S.

Similarly—as will be shown in the section “How the self is modulated”—empirical research that investigates the effects of altering S, even though it allows for the identification of the brain region and neural dynamics that underlie characteristic features of S and for revealing the relationships that hold between the different features of S, falls short of considering and explaining the involvement of CE in the constitution of S. This is because this type of research usually addresses S by adopting theories that explain S in terms of its characteristic features, as they appear in CE (e.g., minimal phenomenal selfhood, sense of agency, and sense of ownership), rather than in terms of how CE contributes to giving rise to these aspects. This leads researchers to consider the characteristic features of S as constitutive elements of S rather than as products of CE. Consequently, they mistake the *explanandum* for the *explanans*, thereby limiting the validity of their research.

To overcome these limitations, it is therefore necessary to resort to theories and models that recognize and account for the role of CE in shaping the sense of S. In the section “Conscious experience and the self,” I review some of the most representative ones (Legrand and Ruby, 2009; Damasio, 2010; Berkovich-Ohana and Glicksohn, 2014; Williford et al., 2018). As will be demonstrated, even though they can account for some of the main aspects of S, they either leave the precise role of CE in the generation of S unexplained or fail to explain it altogether.

As an alternative, I present my model of consciousness (Marchetti, 2022). The model, which still requires empirical verification, is built upon the main assumption that attention is necessary for CE. More precisely, the phenomenal aspect of consciousness is produced by the modulation—engendered by attentional activity—of the energy level of the neural substrate (that is, the organ of attention) that underpins attentional activity.

The main tenet of the model is that the phenomenal aspect of consciousness performs two main functions: it supplies the agent with a sense of S and informs the agent on how its S is affected by the agent’s own operations. The phenomenal aspect of consciousness performs its two main functions through its five main dimensions: qualitative, quantitative, hedonic, temporal, and spatial. Each dimension of the phenomenal aspect of consciousness can be explained by a specific aspect of the modulation of the energy level of the organ of attention.

The five dimensions of the phenomenal aspect of consciousness shape S by bringing about and molding its four fundamental features (the sense of being an entity differentiated from other entities, first-person perspective, the feeling of continuity, and the feeling of unity) and inform on how S is affected by the agent’s own operations.

While not claiming to be exhaustive, my model is, to the best of my knowledge, the first to provide an essential description of how CE—by providing the agent with a sense of S—contributes to building the agent’s S.

## How the self modulates the neural and cognitive processing of stimuli

Most empirical psychological research on S typically focuses on how referring an external or internal stimulus to S biases how the stimulus is processed by processes such as memory, attention, perception, action, reward, decision-making, and emotion (Sui and Humphreys, 2017a). Similarly, neurobiological research investigates how S modulates the neural processing of basic mental functions and higher-order cognitive processes (Northoff, 2016). Let us consider some of these experiments.

Sui et al. (2012b) investigated whether associating a stimulus to S modulates its subsequent perceptual processing. They first required participants to learn associations between abstract geometric shapes (e.g., circle and triangle) and labels that could be either self-related (that is, indicating the participants themselves: e.g., “you”), other-related (that is, indicating a familiar person, e.g., “friend,” “mother,” or an unfamiliar person, e.g., “stranger”), or a neutral word: for example, participants were told: “You are a triangle; your mother is a circle; a stranger is a square.” Afterward, participants were presented with pairs of shapes and labels that either matched or did not match the learned associations. Participants had to judge, as quickly and accurately as possible, whether the presented shape-label pairings matched the learned associations. The experiments demonstrated a substantial advantage (in terms of reaction times and accuracy) of the pairs with a self-related label over the pairs with other-related or neutral labels.

Frings and Wentura (2014) investigated whether the self-prioritization effect found by Sui et al. (2012b) extends from perception-self links to action-self links. In their experiment, participants learned to pair four different movement directions (right, left, up, and down) with four different labels, which could be either self-related (i.e., the pronoun “I”), other-related (“mother” and “stranger”), or neutral: for example, participants were told: “You are the movement to the top. Your mother is the movement to the right. A stranger is the movement to the left. Nothing is the movement to the bottom.” After the learning phase, participants had to execute a movement triggered by a cue with the mouse cursor. Then, one of the four labels appeared at the screen center. Participants had to judge, as quickly as possible, whether this label matched the previously executed movement direction according to the learned movement-direction-label. The experiment revealed the same effect of prioritized processing of self-related matching pairs as Sui et al. (2012b): in matching trials, performance (as indexed by reaction times and accuracy) was significantly better for pairs with a self-related label than for pairs with other-related or neutral labels.

This kind of empirical research is very important for various reasons because it reveals the pervasiveness of self-prioritization (Sui et al., 2012a,b, 2015); helps identify the neural underpinnings of self-related processing, i.e., the processing of a stimulus in relation to S (Northoff, 2016, p. 205); provides indications about the possible functions of S (Sui and Humphreys, 2015, 2017a); helps delineate the theoretical concept of S by defining the relationships between S and the other cognitive structures and processes; last but not least, provides positive evidence of the psychological existence of S (which is not a matter of course for everybody: see, for example, David Hume’s opinion, according to which when we look into the contents of our consciousness, we cannot find any self but just a bundle of perceptions).

However, this kind of empirical research has an inherent limit in the possibility of exhaustively explaining the cognitive and neural processes that are constitutive of S. This is because it uses S as an independent variable and does not investigate how CE contributes to bringing about S. As Klein (2014, p. 3) observes, this kind of research targets S in a particular context (cultural, social, cognitive, synaptic, etc.) rather than S *per se* and what S is that serves as the bedrock of these cultural, social, narrative, etc. instantiations.

As such, this kind of research can certainly help identify the cognitive and neural processes that allow for self-association and self-expansion—that is, the assignment of self-specificity to internal or external stimuli (Sui et al., 2012a,b)—or understand how a person’s S affects their behavior in various contexts, but it cannot explain the processes that are involved in the production of S. At best, it can offer an explanation framed in very generic and broad terms, such as Northoff (2016) “basis model of self-specificity,” which, trying to account for the pervasiveness of S in basic mental functions (perception, action, emotion, and reward) as well as higher-order cognitive functions (memory, attention, meta-representations, etc.),^1^ explains S (and self-related processing) as a basic function of the brain’s intrinsic, spontaneous activity that occurs “prior to and independent of any specific function (sensorimotor, affective, cognitive, social, vegetative)” (Northoff, 2016, p. 214). However, such a kind of very generic explanation lends itself to the criticism that the neural processes and structures that have been associated with S might not be self-specific but might be recruited for other (basic or higher) mental functions as well (e.g., consciousness, thought, etc.; a similar criticism was already raised by Legrand and Ruby (2009) and Christoff et al. (2011) in their extensive review of several neuroimaging studies). Considering, for example, the explanation by Northoff (2016), it can be argued that the brain’s intrinsic, spontaneous activity may be responsible for the production not only of S but also of consciousness (considered in its various levels—wakefulness, drowsiness, REM sleep, etc.—and forms—imaginative, perceptual, recollective, etc.), given that consciousness is as pervasive as S (if not even more pervasive than S, because S can be accessed only through CE). In sum, this kind of very generic explanation usually risks failing to differentiate between the processes and structures that underpin S and those that underpin other functions.

## How the self is modulated

A more promising avenue for research on the cognitive and neural underpinnings of S seems to be offered by those studies that investigate how S can be modulated or altered. Such modulations can be temporally and reversibly induced by a wide range of means, including meditation (Berkovich-Ohana et al., 2013; Jerath et al., 2016; Fingelkurts et al., 2016a,b, 2020; Millière et al., 2018), hypnosis (Kallio and Revonsuo, 2003), perceptual deprivation (Glicksohn and Ben-Soussan, 2020), pharmacological means (Millière et al., 2018; Deane, 2020), induced illusory own-body perceptions (Ionta et al., 2011; Petkova et al., 2011; Blanke, 2012; Pfeiffer et al., 2013), and magnetic brain stimulation (Lou et al., 2004; Luber et al., 2012; Dary et al., 2023). Alterations of S can also occur because of neurological disorders, such as epileptic seizure (Johanson et al., 2008; Blumenfeld, 2011, 2012), psychological and psychiatric disorders, such as xenomelia (Brugger et al., 2013), and during states of consciousness other than conscious wakefulness (Laureys, 2005), such as sleep dreams (Windt, 2010, 2015) and near-death experience (Vanhaudenhuyse et al., 2009; Martial et al., 2020).

For exemplification, let us consider the experiment performed by Fingelkurts et al. (2020). Eight highly experienced meditators were requested to mentally induce states representing either increased (up-regulation) or decreased (down-regulation) sense of three different aspects of selfhood: (a) witnessing agency or “Self,” akin to the sensed “center of gravity” of Velmans (2014) and the phenomenal non-conceptual core of Blanke and Metzinger (2009); (b) body representational-emotional agency or “Me,” akin to the “proto-self” of Panksepp (2005) and Panksepp and Northoff (2009) and the “minimal self” of Gallagher (2000) and Gallagher and Frith (2003); and (c) reflective/narrative agency or “I,” akin to the “narrative self” of Gallagher 2000), the “conceptual self” of Neisser (1991), the “autonoetic self” of Gardiner (2001) and Klein (2016), and the “autobiographical self” of Damasio (1999, 2010).

The meditators’ brain activity was monitored by electroencephalogram (EEG). Electroencephalogram data was complemented by first-person phenomenological reports and standardized questionnaires (focusing on the subjective contents of the three aspects of selfhood).

The instructions for the meditators to attain the desired up-and down-regulated states were as follows: “Me-up state”: “Focus on a sense of the body, on the image of your body, on any tiny sensation your body feels, on the related emotional feelings; sense your position, posture or micromovement; sense yourself centered in this body from which you are experiencing the world.” “Me-down state”: “Focus on a sense of open space without the bodily dimension; concentrate on the absence of the physical body image; try to experience yourself wider, expanded, less physically bounded, bodiless as in out-of-body experience.” “I-up state”: “Focus on reflecting upon yourself; analyze yourself by silently talking to yourself; facilitate the labeling of every self-experience or self-memory you have.” “I-down state”: “Try to accept all experiences without judgment and to refrain from applying evaluative labels such as good/bad, right/wrong, or worthwhile/worthless, and to allow self-reality to be as it is without attempts to avoid, escape, or change it; to slow the train of thoughts.” “Self-up state: “Focus on a non-symbolic, non-linguistic sense of self-presenting being; try to enhance the ability to merge into the self, which is a pure self-referential consciousness, where self is only aware of self.” “Self-down state”: “Try to achieve a feeling like in a contentless dream, though being awake, where one experiences a subjective loss of access to the conscious world as well as of the first-person perspective.”

The main finding of the research is that there is a close causal relationship between three self-referential brain networks (or SRN, also referred to as the default mode network or DMN)—namely the frontal Operation Module,^2^ the right posterior Operation Module, and the left postriot Operation Module—and the three aspects of selfhood (“Self,” “Me,” and “I”), respectively.

Additionally, the neurophenomenological results of this research, as well as of previous research on patients with disorders of consciousness (Fingelkurts et al., 2012, 2016a) and brain damage (Fingelkurts and Fingelkurts, 2017), seem to indicate that, among the three Operation Modules, it is the “Self” Operation Module (anterior subnet of the SRN) that provides the necessary and sufficient basic structural feature for the phenomenological sense of being a self, because a decreased “I” Operation Module and “Me-Operation Module” activity does not necessarily imply a decrease or absence of such a phenomenological sense.

Finally, the research clearly shows that “the complex Selfhood, described in terms of brain SRN dynamics and related phenomenological descriptions, is not a fixed entity *living* on its own, but rather an ongoing emergent property generated by the dynamic interrelation of at least three brain SRN modules that support three phenomenological features (witnessing, self-reflection, and self-embodiment) of Selfhood, which are themselves also complex elements having their own composition” (Fingelkurts et al., 2020, p. 21).

Generally speaking, this type of empirical study is very important because it enables the identification of the brain region and neural dynamics that underlie characteristic aspects of S—such as the “Self,” “Me,” and “I” of Fingelkurts et al. (2020). Moreover, it shows the relationships between the different aspects of S, that is, what distinguishes one from the others, whether and how they dissociate, and so on.

However, this type of empirical study usually addresses S by adopting theories that explain S in terms of its characteristic aspects (or factors, features, modes) as they appear in CE—for example, the minimal phenomenal selfhood^3^ of Blanke and Metzinger (2009) or the sense of agency and sense of ownership of Gallagher (2000)—sense of agency and sense of ownership—rather than in terms of how CE contributes to giving rise to these aspects.^4^ This leads researchers to consider the characteristic aspects of S as foundational, constitutive elements of S rather than as products of CE, thereby mistaking the *explanandum* for the *explanans*. Consequently, this severely hinders or limits the quality and progress of their scientific inquiry.

First, researchers may be led to mistake the neural underpinnings of one or some aspects of S for the neural underpinnings of S *per se*. Consequently, what they claim to be the neural underpinnings of S may turn out to be the neural underpinning of one specific aspect of S. For example, reviewing the empirical findings that seem to support the view that self-related processes are supported by task-negative/default-network regions, Christoff, et al. (2011, p. 4) observe that: “treating self-related processing as the main form of self-experience limits self-experience to the ‘Me’ (self as object of one’s attention) while neglecting the ‘I’ (self as knowing subject and agent).”

Incidentally, the notion that there might be an aspect of S that is more fundamental or basic than others, i.e., the simplest form of S, which accompanies most, if not all, conscious states, is empirically untenable. As Millière (2019) clearly shows, the various aspects (or modes) of S are dissociable, and there is not one mode that is more fundamental than others. Millière identifies three main modes of S: cognitive self-consciousness, bodily self-consciousness, and spatial self-consciousness. His review shows that there can be: (i) spatial self-consciousness without bodily self-consciousness, as evidenced by out-of-body experiences (OBEs), bodiless dreams, and certain drug-induced states that involve a complete loss of bodily awareness, while preserving self-locating content; (ii) bodily self-consciousness without spatial self-consciousness, as exemplified by deafblind subjects, artificial sensory deprivation, and certain meditation practices by means of which expert practitioners are able to achieve altered states of consciousness in which they lack self-locating content, while maintaining a (albeit light) sense of body; (iii) cognitive self-consciousness without bodily self-consciousness, as evidenced by asomatic out-of-body experiences (OBEs), lucid bodiless dreams, and some instances of drug-induced experiences of disembodiment in which subjects do not seem to necessarily impair the ability to have *de se* thought; (iv) cognitive self-consciousness without spatial self-consciousness, as evidenced by extreme sensory deprivation, certain cases of deafblind subjects, and certain drug-induced states in which the awareness of both one’s location in egocentric space and one’s body are suppressed, but *de se* thoughts still occur.

Evidence on the dissociability of the various aspects or modes of S, and, consequently, the untenability of the notion that one aspect or mode of S is more fundamental than others, also comes from experiments on induced illusory own-body perceptions. For example, Ionta et al. (2011) and Pfeiffer et al. (2013) show that self-identification with the body, that is, the experience of owning a body, does not depend on the experienced direction (e.g., upwards vs. downwards) of the first-person perspective.

Second, researchers are led to attribute properties, which actually belong to another process, to aspects of S, thereby undermining their analyses and proposals. For example, Christoff et al. (2011) attribute the property of enabling the distinction between self and non-self to the egocentric perspective. In their view, the “I” (the self as a subjective knower and agent) consists of a self-specific, agentive perspective from which perception and affective processes occur. The agentive perspective would be implemented by basic sensorimotor integration processes and homeostatic regulation processes that systematically couple efferences with their reafferent consequences, thus enabling a functional self/non-self distinction. However, as Vosgerau and Newen (2007) show, this model presupposes the self-world distinction rather than explaining it. Indeed, for the agent to learn the effects of its own movements, it *must already know* which movements are caused by itself and which are not—a knowledge which, in my view, only CE can provide.

The main limitation of the type of empirical study considered in this section, that is, mistaking the *explanandum* for the *explanans*, can only be overcome by duly considering the role of CE in constituting S because it is thanks to CE that S and all its various aspects and modes take form.

Let us then consider some of the works that recognize the role of CE in shaping the sense of S to see if and how they contribute to the advancement of research.

## Conscious experience and the self

Various (philosophical, cognitive, and neuroscientific) theories and models of S have been proposed that, in one way or another, consider CE as a factor in the generation of S: see, for example, Damasio (1999, 2010), Gallagher and Marcel (1999), Zahavi (2000), Legrand (2006, 2007), Legrand and Ruby (2009), Williford et al. (2012, 2018), Marchetti (2012a, 2022), Gallagher (2013), Berkovich-Ohana and Glicksohn (2014), Gallagher and Daly (2018), and Reddy et al. (2019).

According to my analysis, most of them either leave the precise role of CE in the generation of S unexplained or fail to explain it altogether. Let us consider some representative models that, more than others, try to account for the dependence of S on CE, even though they partly fail to do so for the reasons that I will expound.

Damasio is quite explicit in linking CE to S: “Conscious minds arise when a self process is added onto a basic mind process (…) A knower, by whatever name one may want to call it—self, experiencer, protagonist—needs to be generated in the brain if the mind is to become conscious” (Damasio, 2010, pp. 8, 11). Among the foundational elements of S, he gives particular emphasis to the self/non-self distinction, which is made possible by what he calls “primordial (bodily) feelings.” Primordial feelings are spontaneous feelings of the living body: they indicate that “my own body exists, and it is present, independently of any object with which it interacts, as a rock-solid, wordless affirmation that I am alive” (Damasio, 2010, p. 185). According to Damasio, primordial feelings are generated by the “protoself,” which is the basic level of S and serves as the foundation for the other two levels of S: the “core self” and the “autobiographical self.” The protoself is an integrated collection of neural patterns that map, moment-by-moment, the most stable aspects of the organism’s physical structure (Damasio, 2010, p. 190). As such, the protoself provides a reasonably stable platform and source of continuity and singularity, which allows for detecting and registering the variations that the organism undergoes.

Damasio puts forward the hypothesis that the main brain structure that underlies the protoself and, consequently, the generation of primordial feelings is the brain stem. However, as he himself acknowledges, his explanation is not sufficiently comprehensive: “A skeptic may well conclude that I have not answered the question of why feelings feel the way they do, let alone why they feel like anything. Here I both agree and disagree. I have certainly not provided a comprehensive explanation for the making of feelings, but I am advancing a specific hypothesis” (Damasio, 2010, pp. 242–243). He can only observe that primordial feelings “are *felt* images of the body” (Damasio, 2010, p. 188) and have “a definite *quality*, a *valence*, somewhere along the pleasure-to-pain range” (Damasio, 2010, p. 185). He also adds that “Whenever brains begin to generate primordial feelings (…) organisms acquire an early form of *sentience*” (Damasio, 2010, p. 26, italics are mine).

It can be observed that by defining primordial feelings as something that is “felt” or has “a definite quality,” Damasio leaves unexplained not only what it is that makes primordial feelings feel the way they do but also how an organism can feel any feeling (indeed, to be “felt,” primordial feelings must be felt by someone who can feel them). In doing so, he completely overlooks the possible role that the mechanism allowing an organism “to feel” anything may play in generating S, for example, in delimiting the borders of the organism and consequently in differentiating the self from the non-self. As Gallagher observes, it is consciousness that enables an organism to experience the difference between itself and others: “*To the extent that the bodily system can be conscious*, it will pre-reflectively experience, from a first-person perspective, the self/non-self distinction in the various sensory-motor modalities available to it (e.g., kinesthesia, proprioception, touch, vision). Such aspects contribute to an experiential and embodied sense of ownership (…) and a sense of agency for one’s actions” Gallagher (2013, pp. 3-4, italics are mine).

In sum, Damasio’s proposal, even if it acknowledges the close link between CE (the “primordial feelings”) and S (“The self would consist of the primordial feelings that the protoself, in its native state, spontaneously and relentlessly delivers, instant after instant”; Damasio, 2010, p. 202), fails to show how this link works.

In her works, Legrand also acknowledges the close link between CE and S: “Anybody who denies the subjectivity of experiences and considers that experiences can be given in a neutral way simply fails to recognize an essential aspect of what it feels like to undergo an experience” (Legrand, 2007, p. 585).

According to Legrand and Ruby, what makes experiences my own experiences is the specific “perspective” that characterizes them: “My perceptions, representations, and experiences are anchored in my perspective, and by virtue of this, they are mine rather than someone else’s or nobody’s. In this view, being a self (…) corresponds to experience the world from one’s specific perspective” (Legrand and Ruby, 2009, p. 274). It is perspective that constitutes the self/non-self distinction: “The perspective is fundamentally a self-specifying process in the sense that it constitutes the self–non-self distinction. The self is differentiated from non-self in a systematic manner, thanks to the fact that a perspective relates self and non-self in a nonsymmetrical manner: The self is representing, and the non-self is represented” (Legrand and Ruby, 2009, p. 275).

In addition to highlighting the importance of perspective for the delimitation of S, Legrand and Ruby acknowledge the essential role that perspective has for CE: “A perspective grounds every perception and representation held by any given subject (…) the constitution of the perceiving self and its perspective are concomitant to the perceptual act” (Legrand and Ruby, 2009, pp. 274, 275). They also specify that subjective perspective is made possible by processing a given perceptual content “according to the fact that it is a reafference, that is, according to the fact that it is related to oneself as a perceiving agent/active perceiver. Our proposal is that relating an efference with its reafference is a process enabling the perceptual act to be characterized not only by a given content (the acidity of the lemon) but also by a self-specific perspective (I am the one experiencing the acidity of the lemon juice)” (Legrand and Ruby, 2009, p. 277). At a basic physiological level, this process: “is grounded in sensorimotor integrative processes relating efferent information to its reafference and allowing any represented object to be related to the representing subject” (Legrand and Ruby, 2009, p. 278).

However, Legrand and Ruby do not proceed forward in investigating, first and foremost, how it is possible for an organism, before relating any object to itself, to become conscious of anything. Without performing such a preliminary analysis, they, like Damasio, overlook the possible role that the mechanism enabling an organism to have CE may play in generating S, especially in differentiating the self from the non-self, particularly when non-strictly physical or biological dimensions are involved, such as the psychological or social ones. Indeed, if “the constitution of the perceiving self and its perspective are concomitant to the perceptual act,” as Legrand and Ruby (2009, p. 275) recognize, then it can be claimed that perspective is brought about by the conscious act and that the self/non-self distinction is the product of the conscious act rather than of perspective.

Williford et al. (2018) put forward an explanation of how their model of consciousness—the Projective Consciousness Model—can account for some of the main aspects of S, namely, the first-person point of view, pre-reflective self-consciousness, global self-consciousness, social self-awareness, ipseity or “mineness” (foundational to the sense of body ownership and agency), the “transcendental ego,” and the autobiographical self or “empirical ego.”

The Projective Consciousness Model is intended to show “how consciousness allows organisms to integrate multimodal sensory information, memory, and emotion in order to control behavior, enhance resilience, optimize preference satisfaction, and minimize predictive error in an efficient manner” (Williford et al., 2018, p. 2). To this end, the Projective Consciousness Model adopts a combination of two models: a model of perspective-taking based on 3D projective geometry (“Field of Consciousness”), which embeds a point of view, and a model based on the principles of active inference driven by Free Energy minimization (Friston, 2010). The former model accounts for the perspectival characteristics of CE: the perceived or imagined world appearing to the organism is three-dimensional and perspectival, unfolding in an oriented manner between a point of view and a horizon at infinity, where all parallel lines converge; the origin of the point of view, which functions as an innermost zone around which experience is organized, is elusive; the space is normally organized around the lived body. The latter model accounts for an organism’s activity to minimize predictively erroneous representations of the world to maximize the accuracy of its beliefs and the satisfaction of its preferences in a globally optimal manner (an organism performs this activity by predictively anticipating the consequences of its actions, programming its actions accordingly, and updating its prior beliefs based on a comparison between its predictions and sensory evidence to minimize predictive error. Cycles of perception, imagination, and action, as well as prior updates, are used to minimize Free Energy).

According to Williford, et al. (2018, p. 8), the combination of the two models allows for accounting of how an organism can optimize the precision of its knowledge about the causes of its sensations and the satisfaction of its preferences based on the Field of Consciousness, which frames the distribution of Free Energy attached to objects and affordances across space and time, factually or by anticipation.

As observed before, the Projective Consciousness Model can account for some of the main aspects of S. For example, the “transcendental ego” can be accounted for by the fact that the origin of projection of the projective 3D space cannot appear in the Field of Consciousness as an object in the space, but it can only appear elusively, encoded in the geometrical structure of the Field of Consciousness as a fourth dimension. As Williford et al. (2018, pp. 12–13) explain: “The origin cannot be experienced as a perceptual object. In that sense, it could never look at itself or catch its tail. In fact, there is no ‘thing’ here that could ‘look at itself,’ but rather a virtual pivotal point essential for the rendering of lived space, and necessary for establishing a direction of aim or ‘vectorization’ of the Field of Consciousness.”

Despite all its insights, the Projective Consciousness Model does not address the fundamental question of how it is possible for an organism to feel anything. The Projective Consciousness Model explains why things appear differently when observed from a different perspective but not what makes an organism perceive or feel any appearance at all. Williford, et al. (2018, p. 8) limit themselves to observing that vision is “like a ‘palpation’ of a 3D spatial ‘user surface’ to ‘feel’ the epistemic affordances that are quantified by expected Free Energy” but do not explain the mechanisms and processes that make this “feeling” possible. Similar to Damasio and Legrand and Ruby, this leads Williford et al. to overlook the role that the mechanism underlying CE plays in differentiating the self from the non-self. In fact, the Projective Consciousness Model does not explain how the distinction between self and non-self takes place, but rather it presupposes it by assuming the relational character of CE: “Consciousness seems to involve the perspectival presentation of something (an object, quality, or state of affairs) to the conscious system” (Williford et al., 2018, p. 3). That is, the mere existence of a point of view or perspective would be a sufficient, motivating reason for the self/non-self distinction.^5^ However, as evidence shows (Millière et al., 2018; Millière, 2019), there are cases in which spatial self-location and point of view can disrupt, but *de se* thoughts still occur.

Not addressing the question of how the self/non-self distinction occurs severely limits the capacity of the Projective Consciousness Model to explain how CE contributes to giving rise to S.

The model of Berkovich-Ohana and Glicksohn (2014)—the Consciousness State Space model—describes the close links between CE and S. They suggest that all CEs can be classified into two main categories: Core Consciousness, the scope of which is the here-and-now, and Extended Consciousness, which involves memory of past, the imagination of future, and verbal thought. Core Consciousness and Extended Consciousness each support the two main aspects of S, namely the Minimal Self and the Narrative Self, respectively. The Minimal Self is endowed with a sense of agency, ownership, and non-conceptual first-person content, and the Narrative Self involves personal identity and continuity across time, as well as conceptual thought. Importantly, Extended Consciousness is always dependent on Core Consciousness, and the Narrative Self is always dependent on the Minimal Self, not vice versa. The Consciousness State Space is a phenomenological space articulated along three main dimensions: time, awareness, and emotion. Each of the three dimensions has a dual phenomenological composition, falling within Core Consciousness and Extended Consciousness.

Their model offers a detailed review of the (attentional, memory, cognitive, etc.) processes that may underlie the three main dimensions of the Consciousness State Space. It also offers a detailed *description* of how the structure of each dimension may be reflected in the structure of S and its two main aspects, Minimal Self and Narrative Self. However, their model does not *explain* the mechanisms and processes that allow CE to bring forth S and its characteristic aspects. Moreover, it fails to address how the self/non-self distinction can take place. Instead of explaining how Core Consciousness and Extended Consciousness generate the Minimal Self and Narrative Self, respectively, they proceed straightly to discuss the plausible neural underpinnings of the Consciousness State Space. In my view, this choice is quite contradictory because I do not see how one can identify a neural substrate of any process (or function) without having first identified the process itself.

To overcome all these limits, I have developed a model (Marchetti, 2022; for previous versions, see: Marchetti, 2010, 2012a, 2018) on how CE contributes to shaping the sense of S. The model, which requires empirical verification, is built upon the most important assumption that attention is necessary for CE. This assumption is based on various empirical evidence. First, there are experiments demonstrating that attention modulates perception, directly influencing how we consciously experience the world. Psychological studies of visual perception provide empirical evidence that attention alters phenomenal appearance (Carrasco et al., 2004; Liu et al., 2009; Carrasco, 2011; Barbot et al., 2018). Psychological studies of the perception of time show that attention modulates perception: for example, the prior-entry effect shows that when one attends to a stimulus, one perceives it as having occurred earlier than if one was not attending to it (Shore et al., 2001); experiments on duration judgments show that a higher amount of attention allocated to the passage of time itself produces a lengthening of the experienced duration (Hicks et al., 1976, 1977; Brown, 1985; Coull et al., 2004). Inattentional Blindness experiments (Mack and Rock, 1998) show that there is no explicit, conscious perception before the engagement of focal attention. Change Blindness experiments reveal that under flicker conditions (Rensink et al., 1997), which alter the allocation of attention, observers fail to notice major changes even when changes are large and made repeatedly.

Second, attention and CE share important features. Both attention (VanRullen et al., 2007; Bush and VanRullen, 2010; Landau and Fries, 2012; Fiebelkorn et al., 2013; Song et al., 2014; Zoefel and Sokoliuk, 2014; Dugué et al., 2015; VanRullen, 2016, 2018; Fiebelkorn and Kastner, 2019; Nakayama and Motoyoshi, 2019; Senoussi et al., 2019; Zalta et al., 2020) and CE (Kranczioch et al., 2007; Van Dijk et al., 2008; Busch et al., 2009; Doesburg et al., 2009; Mathewson et al., 2009; Fingelkurts et al., 2010; Romei et al., 2010; Neuling et al., 2012; Blais et al., 2013; Wutz and Melcher, 2014; Baumgarten et al., 2015)^6^ operate in a periodic, pulse-like manner, and, importantly, attention critically shapes conscious perception (Dugué and VanRullen, 2017; Nakayama and Motoyoshi, 2019; Gaillard and Ben Hamed, 2022).

Moreover, both attention and CE share an egocentric spatial organization. As Merker (2013, p. 9-10) observes, attention is deployed from a single point, which is located inside our body, namely “at the proximal-most end of any line of sight or equivalent line of attentional focus.” This point “is excluded from the contents of consciousness by the same geometric necessity that prevents an eye from viewing itself, though it is the instrument for viewing all else” (Merker, 2013, p.10). Similarly, our CEs are spatially arrayed around a central point egocentrically defined by our body and can be localized relative to it. This point constitutes the center of a reference system that defines the space in which all the objects of CE are located. Even the most abstract CEs are spatially localized: our emotions are located somewhere in our body; we feel our memories as originating and located in ourselves; we have ideas and thoughts in our mind, etc. (Revonsuo, 2006). Importantly, as Williford et al. (2012, 2018) observe, this point is “elusive” in that it is not located within the space of consciousness itself but outside the phenomenal conscious space.

Two clarifications are in order here. First, even though attention is necessary for CE, attention and CE are different processes: indeed, there can be attention without CE (Naccache et al., 2002; Montaser-Kousari and Rajimehr, 2004; Sumner et al., 2006; Bahrami et al., 2008). Second, the debate is still open about whether attention is really necessary for CE (see, for example, Maier and Tsuchiya, 2021). However, as already observed by various scholars (Srinivasan, 2008; Kouider et al., 2010; Marchetti, 2012b; Pitts et al., 2018; Munévar, 2020; Noah and Mangun, 2020), the claim that attention is not necessary for CE seems to result from a wrong interpretation of the experimental data, which originated from not having considered the various forms and levels that attention (e.g., bottom-up, top-down, focused, and diffused) and CE (e.g., anoetic, noetic, and autonoetic) can assume (e.g., not all forms of attention produce the same kind of CE, and not all forms of CE are produced by the same kind of attention; there can be kinds of CE with no top-down attention but with bottom-up attention; there can be kinds of CE in the absence of a focal form of top-down attention but in the presence of a diffused form of top-down attention).

I summarize the main tenets of my model that are most relevant to what is being discussed here, providing supporting evidence and/or conceptual premises for each:^7^

- What primarily distinguishes CE from other phenomena is its phenomenal aspect, that is, the what-it-is-like for an agent to experience something (Nagel, 1974).
- The phenomenal aspect of consciousness has two main functions: to supply the agent with a sense of S and inform the agent on how its own operations affect its S.
- The phenomenal aspect of consciousness is produced by attentional activity, which modulates the energy level of the neural substrate (that is, the organ of attention) that underpins attentional activity. The raw material for attention is derived from information that is endogenously generated by the agent (e.g., interoceptive stimuli, memories, and mental images) or coming from the external environment (e.g., exteroceptive stimuli, speeches, and written texts). Usually, but not always, attentional activity selects and enhances information that is physically salient or most relevant for the agent’s current goals or selection history or for the maintenance of the agent’s homeostatic values.
  The search for the organ of attention has been ongoing for many years (e.g., Mesulam, 1990; Posner and Petersen, 1990; Crick and Koch, 2003; Vossel et al., 2014; Benedek et al., 2016; Yeager et al., 2021) and is not fully uncontroversial (De Brigard, 2012): some scholars suggest that there may not be a single neural process responsible for all forms of attention (Wu, 2011), while others suggest that attention is a unified cognitive process with an identifiable sub-personal neural correlate (Prinz, 2011). It goes without saying that only a clear definition of the features and functions of attention can help define the nature of its organ. In my view, the model of attention that best aligns with my conception of an organ of attention is by Tamber-Rosenau and Marois (2016). They conceptualize attention as a structured mechanism arranged at various levels and parts with different functional roles: a central level for abstract, cognitive processes, a mid-level containing priority maps that bias competitions in representational formats and sensory modalities, and a peripheral level for sensory processes. According to this model, the organ of attention can be conceived as structured at various levels and parts, each underpinning these different roles.
  The hypothesis that attentional activity produces a modulation of the energy level of the organ of attention is based on the observation of the extreme consequences that such modulation can bring about. An example of this is the sensation of pain, which primarily consists of an interruption of the normal flow of attention (Eccleston and Crombez, 1999; Legrain et al., 2009).
- The phenomenal aspect of consciousness is characterized by five main dimensions: qualitative (the what-it-feels-like of an experience: e.g., what it feels like “to see red” vs. “to smell garlic”), quantitative (the intensity of an experience), hedonic (the pleasantness, unpleasantness, or indifference of an experience), temporal (the duration of an experience), and spatial (the egocentric perspective in which an experience is embedded).
  This characterization of the phenomenal aspect of consciousness partly follows Cabanac (2002), who, however, does not include the spatial dimension.
- Each dimension of the phenomenal aspect of consciousness can be explained by a specific aspect of the modulation of the energy level of the organ of attention. (i) The qualitative dimension is defined by the specific area of the organ of attention that underpins and is consequently modulated by attentional activity. This hypothesis derives from the conception of the organ of attention as an organ structured at various levels and parts, each underpinning the different functions of attention. (ii) The quantitative dimension is defined by the amplitude of the modulation. This hypothesis relies on the evidence that attention can be applied at variable levels of intensity (Kahneman, 1973; La Berge, 1995) and that stimuli can be of various intensities: the combinations of these two factors engender different modulations of the energy level of the organ of attention. (iii) The hedonic dimension is defined by the direction of the modulation, that is, whether the energy level of the area of the organ of attention moves toward or away from the set-point at which the energy level of the area is set. In this view, pleasant and unpleasant experiences occur when the energy level moves toward or away from the set-point, respectively. Neutral experiences—a state characterized by physiological normality and indifference toward the environment—occur when the energy level fluctuates within an acceptable range of the set-point. The hypotheses of the set point and its adjustability are derived from Cabanac (1971, 1979, 2006) and Cabanac and Russek (2000). A similar explanation of the hedonic dimension in terms of deviations to and from a set-point is also put forward by Solms (2019). (iv) The temporal dimension is determined by the periodic nature of the attentional activity—a nature that limits the duration of the modulation and, consequently, of any CE. This limit is overcome primarily with the support of working memory. (v) The spatial dimension is determined by the egocentric spatial nature of attention.^8^ Attention originates from a single point located inside our body and is directed toward something. The path that attention takes is reflected in the path of the modulation of the area of the organ of attention. The egocentric perspective constitutes the original framework upon which the allocentric perspective can be built, relying on additional capacities such as translocating the egocentric perspective to external objects, creating spatial maps, remembering spatial information, and integrating spatial and visual information. These capacities require the support of additional cognitive mechanisms such as working memory and procedural memory.
- The sense of S supplied by the phenomenal aspect of consciousness is characterized by some fundamental features, the main ones being the following: (i) The sense of being an entity differentiated from other entities. This sense provides the agent with a sense of mineness or ownership; (ii) The point of view or perspective from which any content is experienced; (iii) The feeling of continuity, that is, that our experience flows uninterruptedly; and (iv) The feeling of unity or a “single voice” (Damasio, 2010), that is, of being an organism composed of multiple parts interconnected in a unified way. These features do not always need to be present together at the same time. Depending on the arousal state—e.g., conscious wakefulness, REM sleep, vegetative state, and near-death experience (Laureys, 2005)—and the modes of self-consciousness—e.g., spatial, bodily, or cognitive self-consciousness (Millière et al., 2018; Millière, 2019)—some of them can be altered or even missing altogether.
- Each of the main features of S is shaped by one or more of the five dimensions of the phenomenal aspect of consciousness. The sense of being an entity differentiated from other entities is primarily made possible by the hedonic dimension, perspective by the spatial dimension, the feeling of continuity by the temporal dimension, and the feeling of unity by the concurrent support of the qualitative, quantitative, temporal, and spatial dimensions. A clarification is in order here. My claim that the phenomenal aspect of consciousness supplies the agent with a sense of S implies a sort of identity between the phenomenal aspect of consciousness and S, suggesting that all CEs are subjective. Someone could reject my claim by arguing that, in some cases, CE does not give rise to a sense of S. Consider, for example, the alleged cases of self-loss or ego-dissolution reported by highly experienced mindfulness meditators: “it’s like falling into empty space… and a sense of dissolving […] there’s no personal point of view, it’s the world point of view, it’s like the world looking, not [me] looking, the world is looking” (Millière et al., 2018, p. 11), or by users of psychedelic drugs: “I wasn’t anything anymore. I had been broken down into nothingness, into oblivion” (Millière, Carhart-Harris et al., 2018, p. 16). My response to this argument is—following Gallagher (2017)—that it is not clear how one can report on these extreme states of CE without having first recognized them as one’s own (and not as someone else’s). Furthermore, it can be added that it does not matter whether the “one” these states refer to is myself, the world, the universe, everything, or even nothing, or whether it implies a perspective centered onto a single point of origin inside one’s body rather than a perspective from everywhere or nowhere. To be able to say that “I was the universe, I was everywhere and nowhere” or “(I forgot) that I was a male, a human, a being on Earth—all gone, just infinite sensations and visions” (reported by Millière et al., 2018), one must have been aware, while experiencing those extreme experiences, that they were experienced by oneself, whatever “oneself” refers to at the time of the experiences. Therefore, in my view, it is legitimate to conclude that CE always implies at least a minimal level or form of S, even if some of its features can be missing.
- The information on how the agent’s S is affected by the agent’s own operations can be provided by any of the five dimensions of the phenomenal aspect of consciousness. The most relevant information is provided by the hedonic dimension because it indicates how much the energy level of the organ of attention departs from the set-point (at which the level of the area of the organ of attention is set), thereby signaling what falls and what does not fall under the control of the agent. For example, the experience of pain informs the agent that it is undergoing a specific variation that affects it as a single unit and that this variation hinders or blocks the agent’s capacities.
  Typically, the agent’s operations induce temporary modulations of the energy level of the organ of attention, consequently causing temporary variations in the state of S. However, there are instances where these operations have no effects, which may lead the agent to think or say that “nothing happened” or “I see no difference.” These temporary variations or absence of variations provide the agent with immediate and intuitive knowledge (on which rational knowledge can subsequently be developed) of how entities and events relate to the agent’s S. Moreover, they allow the agent to progressively develop its S. Indeed, S is not simply given but must be learned and achieved (Rochat, 2003; Cleeremans, 2008; Ciaunica et al., 2021) through the continuous process of differentiation—primarily fostered by CE—between the agent and the other entities. Conversely, the continuous development of S leads to changes in how the individual experiences the same event or object: for example, repeated processing of a stimulus leads to habituation, and repeated practice to automatization of the practiced skill (Baars, 1988); experience of duration changes with an individual’s age (Flaherty, 1999).

## Conclusion

The primary determinant of our intuition of S is CE. Without CE, it would be impossible for S to be described and take on all its features. Despite this, empirical research on S does not tackle the problem of how CE contributes to building S. In this article, we have seen that usually, empirical research investigates how S either biases the cognitive processing of stimuli or is altered through a wide range of means (meditation, hypnosis, neurological disorders, etc.). In the former case, empirical research uses S as an independent variable and does not investigate how CE contributes to bringing about S: this limits the possibility of exhaustively explaining the cognitive and neural processes that constitute S. In the latter case, empirical research usually investigates one or some characteristic aspects of S. However, because the theoretical models it adopts do not embed the notion that S is primarily shaped by CE, it assumes that these limited aspects are foundational, constitutive elements of S, thereby mistaking the *explanandum* for the *explanans*. This main limitation unavoidably undermines the research’s validity.

Even the majority of the models that consider CE as a factor in the generation of S leave the precise role of CE in the generation of S unexplained or fail to explain it altogether. This usually happens because they lack a plausible explanation of how an organism can be conscious of anything. Because of this, they overlook the role that CE plays in generating S, specifically in differentiating the self from the non-self.

To overcome these limitations, it is necessary to develop a model that describes how CE contributes to generating S. This is the aim of the model that I have put forward (Marchetti, 2022). The model offers the conceptual advantage of accounting, through a single mechanism, for how CE contributes to giving rise to the various forms that S can take on. This means that it is possible to explain the various forms of S as outcomes of the various ways in which attention may (be made to) operate, and the energy level of the organ of attention may be modulated.^9^ Let us compare, for example, the sense of S elicited by near-death experience with the sense of S elicited by normal conscious awake states. Near-death experiences usually occur in patients who are in transitory and reversible cardiac arrest. The sense of S elicited by near-death experience may imply, among other aspects, out-of-body experiences (OBE) and a panoramic life review (Vanhaudenhuyse et al., 2009; Martial et al., 2020). If we hypothesize that the organism’s supplied energy to the organ of attention determines the agent’s state of wakefulness and that the supplied energy can vary according to various factors such as cardiac arrest, drug use, and the agent’s expectations, we can explain—in line with Martial et al. (2020)—the specific sense of S elicited by near-death experience as resulting from a low energy supply, which is nevertheless sufficient for the organ of attention to support some minimal internally (i.e., disconnected from the environment) deployed attention. In contrast, the sense of S elicited by normal conscious, awake states results from a standard energy supply that can support both internally and externally deployed attention.

In this view, given the close link among attention, CE, and S, and considering that there can be attention without CE, it should be of interest to test whether and to what extent attention alone (without CE) can modulate S.

The model also offers the methodological advantage of providing a unifying theoretical framework for researchers who intend to identify the neural underpinnings of S. By describing the mechanism that underlies the construction of S *per se*, the model indicates what researchers must look for when they investigate the neural processes and structures supporting S and its various actualizations. Current empirical research on the neural underpinnings of S is prevalently conducted by inferring them from experimentally induced or pathological alterations in *partial* aspects of S. This approach yields a fragmented and sometimes (apparently) contradictory representation of the neural structures and processes underlying S, which only a unifying theoretical framework such as the one I have put forward can integrate.

Finally, the model sheds new light on the mechanisms that allow CE to shape S. Let us consider, for example, the role and power of language. It is known that the grammatical class of nouns can help shape an individual’s identity by giving them the possibility to identify with a certain category of persons (Carnaghi et al., 2008; Bryan et al., 2014) or to avoid identifying with a socially undesirable, negative behaviors (Bryan et al., 2013). For example, as the experiments of Bryan et al. (2014) show, 3–6-year-old children are more willing to assist adults with chores (e.g., picking things up) when they are referred to as “helpers” rather than when they are asked “to help”: requesting them to take on the role of a helper results in nearly a 30% increase in their willingness to assist, as opposed to simply asking them to help. As Berger (2023, p. 21) observes, turning a verb (“help”) into a noun (“helper”) turns “what was previously just an action (i.e., helping) into something more profound. Now picking up blocks is not just helping, it’s an opportunity. An opportunity to claim a desired identity.” The model that I propose allows for accounting for how language helps shape S. Considering that language is a tool to pilot attention (Ceccato and Zonta, 1980; Marchetti, 2006, 2023; Benedetti, 2011; Marchetti et al., 2015) and that S is primarily determined by CE, which is brought about by attentional activity, the avenue is opened to analyze how language can affect S.

## Author contribution

GM: Writing – original draft.

## Footnotes

^1^See also Scalabrini et al. (2022), where S is considered as a “psychological baseline or prior” on the basis of which incoming signals are processed.

^2^Operational Modules are short-term metastable topological combinations of sequences of segments between different electroencephalogram channels, which are to a certain extent synchronized (Fingelkurts et al., 2010; Fingelkurts and Fingelkurts, 2011).

^3^The central defining features of minimal phenomenal selfhood are as follows: (i) a globalized form of identification with the body as a whole (as opposed to mere ownership for body parts), (ii) spatiotemporal self-location, and (iii) a weak, geometrical first-person perspective (Blanke and Metzinger, 2009).

^4^Some of these theories put forward an explanation of the possible mechanisms and processes underpinning the aspects of S that they address: see, for example, Gallagher’s (2000) forward and feedback comparators, the comparator mechanism for relating efferent signals to reafferent sensory feedback of Christoff et al. (2011), and Seth’s (2013) interoceptive inference. However, it must be noted that these mechanisms and processes are generally designed for broad purposes and are not specifically intended to explain CE. Therefore, these theories cannot be strictly considered as addressing how CE constitutes S.

^5^See also Williford et al. (2012, p. 348): “The same medium and mechanisms that constitute the self-perspective intrinsic to the projective space naturally generate a basic intentional relation, understood in a minimal sense as a directionality.”

^6^However, it should be noted that this field of research is not immune to controversy. See, for example, Fekete et al. (2018) and Keitel et al. (2022).

^7^Please refer to Marchetti (2022) for a detailed account of the model, its main theoretical and empirical assumptions, and the possibilities of its empirical verification.

^8^It is important to highlight that the spatial dimension of the phenomenal aspect of consciousness must not be confused with the experience of space. One can consciously experience something without being aware of the spatial dimension of one’s experience. The spatial dimension of the phenomenal aspect of consciousness is a precondition for any experience to occur, including the experience of space, but it is not in itself an experience of space. For an experience of space to occur, a specific assembling—performed with the support of working memory—of the contents selected by attention is necessary (Marchetti, 2014). The same holds for the temporal dimension.

^9^Attention may not always be the sole determinant, and additional processes may be needed, such as multisensory integration of bodily signals in changes in self-identification (Blanke, 2012).
